# Genome-wide analysis of lectins in cyanobacteria: from evolutionary mode to motif patterns

**DOI:** 10.1186/s12864-023-09790-8

**Published:** 2023-11-16

**Authors:** Tongli Xu, Yulin Cui, Song Qin, Yin-Chu Wang

**Affiliations:** 1 Shandong University of Traditional Chinese Medicine, Jinan, 250335 China; 2 Yantai Institute of Coastal Zone Research, Chinese Academy of Sciences, Yantai, 264003 China; 3 Binzhou Medical University, Yantai, 264003 China; 4 Center for Ocean Mega-Science, Chinese Academy of Sciences, Qingdao, 266071 China; 5 National Basic Science Data Center, Beijing, 100190 China

**Keywords:** Lectin, Gene family, Evolution, Cyanobacteria, Motif

## Abstract

Lectins are glycoproteins that can bind to specific carbohydrates, and different lectin families exhibit different biological activities. They are also present in the cyanobacteria and many of them have shown excellent therapeutic effect, which deserve for bioprospecting. However, in comparison to those from terrestrial plants, the current knowledge on cyanobacterial lectins is very limited. To this end, genome-wide analyses were performed to find out their evolutionary mode and motif patterns in 316 genomes of representative taxa. In results, 196 putative cyanobacterial lectins were dig out and 105 of them were classified into known families. Seven lectins were found to be belonged to distinct two lectin families, and they may have the potential activities of both lectin families. Whereas no MFP-2, Chitin, and Nictaba family lectins were found. What’s more, the Legume lectin-like lectin family was found to be the richest and most complex in cyanobacteria, which could be a main research direction for future cyanobacterial lectin bioprospecting and development. Our classification and prediction of cyanobacteria lectins is expected to provide assistance in the development of lectin-based medicine and provide solutions to the current thorny viral and tumor diseases in humans.

**Supplementary Information:**

The online version contains supplementary material available at 10.1186/s12864-023-09790-8.

## Background

Lectins are glycoproteins of non-immune origin that bind to specific carbohydrate structures and have the function of agglutinating cells or precipitating glycoconjugates [1]. Lectins are found in various organisms, including plants, animals, fungi and bacteria, and even in viruses and mycoplasmas [2]. As prokaryotic bacteria, cyanobacteria are also known to contain lectins [3]. Lectins are not only widely distributed, but also have a very prominent pharmacological value [4]. For example, in terms of antitumor, among the three lectins isolated from mistletoe, ML-I showes a more pronounced activity compared to the other two, exhibiting a wide range of antitumor effects [5]. And clonally expressed ML-I produced additional inhibitory effects on tumor cells in the adjuvant treatment of colon cancer patients in the first phase of clinical trials and was 5000 and 1500 times more potent compared to the current available anticancer drugs adriamycin and paclitaxel, respectively [5]. In the fight against COVID-19, one of the most representative red algal lectins, Griffithsin (GRFT), which is derived from the red alga *Griffithsia* sp., contains 121 amino acids and has a size of 12.7 kDa [6]. And it is a β-prism I structure [7]. Its most representative activity is the inhibition of HIV and SARS-CoV-2, and because of its inhibition of a variety of SARS-CoV-2. Q-GRFT intranasal spray has been developed to prevent COVID-19 and is now in clinical trials as a new drug [8]. In the fight against AIDS, cyanobacterial lectins have shown a more prominent role in fighting HIV compared to terrestrial plant lectins. Both cyanobacterial lectins, Cyanovirin-N (CV-N) and Microcystis viridis lectin (MVL), contain two mannose binding sites. At nanomolar concentrations or lower, they can inhibit HIV-1 envelope-mediated membrane fusion, thereby exerting antiviral effects [9, 10].

Compared with numerous researches on terrestrial plant lectins, researches on algal lectins remain insufficient (including cyanobacteria in this paper). For instance, there are less than one hundred reports on cyanobacterial lectin, mostly focused on MVL and CV-N [2]. There are two reasons for this phenomenon. First, algal lectins were studied almost 80 years later than plant lectins. Since the first plant lectin discovered in 1888, numerous studies on plant lectins have been conducted, and thousands of lectins have been isolated to date [11]. On the other hand, the presence of hemagglutinins in seaweeds was firstly reported in 1966 [12]. Second, algae are usually more difficult to obtain sufficient biomass due to seasonal and geographical limitations compared to many terrestrial plants.

Although the research and development of algal lectins is relatively tardy, it has shown great medicinal potential [13]. Compared to terrestrial plant lectins, algal lectins are mostly monomers, which are not only small in size and low in molecular weight, but also contain disulfide bonds as well as a large number of acidic amino acids [14–16]. Their activity also does not require stimulation by metal ions, are highly stable and antigenic, and are highly specific for oligosaccharides [13], making them more suitable for development as drugs. Currently, lectins are classified into according to their molecular backbones [7]: (1) Griffithsin lectin family (β-Prism I scaffold); (2) OAAH lectin family (β-Barrel scaffold); (3) Bean lectin family (β-sandwich scaffold); (4) GNA-like lectin family (β-Prism II scaffold); and (5) MFP 2 lectin family (MFP 2-like scaffold). They can be classified according to the type of sugar binding of lectins: D-Galp family, D-Manp family, D-GlcpNAc family, etc. And they can be classified according to evolutionary and structural relevance: β-trefoil family, β-helix family, β-Prism II family, etc. [17]. Lectins in the same family are similar in terms of biological activity [7]. The OAAH family, for example, has the same scaffold as the *Oscillatoria agardhii* agglutinin lectin OAA, the β-barrel scaffold [18]. Usually having a similar structural domain tandem repeat structure consisting of about 67 amino acids and divided into two types, i.e. four repeats and two repeats [19], KAA-2 [20], SFL-1 [21], EDA-2 [22], ESA-1 [23], and ESA-2 [24] of the OAAH family shows low concentration activity in both antitumor and antiviral. The attribution of lectins crosses over in various taxonomic approaches, which may predict a balancing of the properties of the two families.

Cyanobacteria, also called blue-green algae, is a diverse group of oxygenated photosynthetic prokaryotes commonly found in a wide range of aquatic and terrestrial environments. Both cyanobacterial and algal lectins are commonly discovered and applied in medical uses [2]. Due to their similar habitat and well-known evolutionary relationship because of massive endosymbiotic gene transfer, the prokaryotic cyanobacteria and other eukaryotic algae are often mentioned together when discussing lectins [13, 25]. Like the conservation of particular domains and several motifs found in lectins highlights algal significance for plants [26], the genomic investigation into cyanobacterial lectins would help better understand and develop lectins from these groups. However, the progress on cyanobacterial lectin research lags behind the research on other algal lectins. According to a statistics of algal lectins in biomedical applications, cyanobacterial lectins account for only 17%, while lectins from green algae and red algae account for 22% and 61%, respectively [13]. Bioinformatic analysis is a powerful analytical tool, which was used by van Holle and van Damme [27] to analyze the origin and evolution of lectins in plants. Dang et al. [28] and Eggermont et al. [29] made an analysis of the presence of lectins in cucumber and the model plant *Arabidopsis*, respectively, which is useful for understanding the potential biological activity of lectins in cucumber and *Arabidopsis* and helps in the exploitation of lectins. Likewise, the available amino acid sequence data of cyanobacterial lectins and the genomic and transcriptomic data of cyanobacteria were collected to analyse the distribution and evolution of cyanobacterial lectins. This will facilitate the chemical synthesis and heterologous expression of algal lectin peptides and provide a basis for the discovery and activity studies of cyanobacterial lectins.

## Results and discussion

### Taxonomic distribution of lectin domains in cyanobacteria

**Fig. 1:**
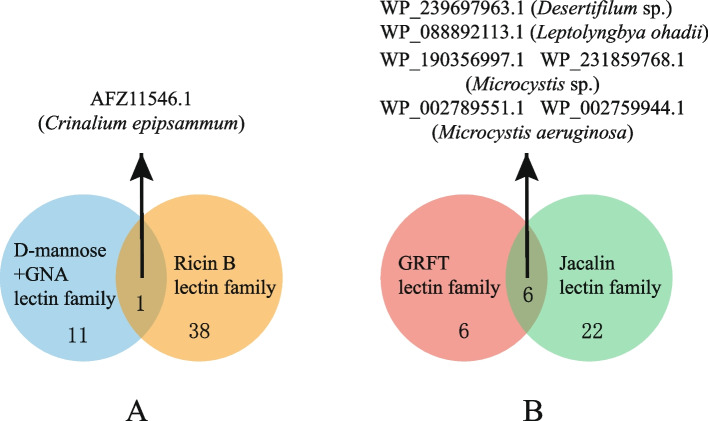
Venn diagrams of cyanobacterial lectins with domains belonging to two different families. **A** One lectin from *Crinalium epipsammum* with domains of D-mannose + GNA family and Ricin B family; **B** Six lectins from *Microcystis aeruginosa* et al. with domains of GRFT family and Jacalin family

**Fig. 2:**
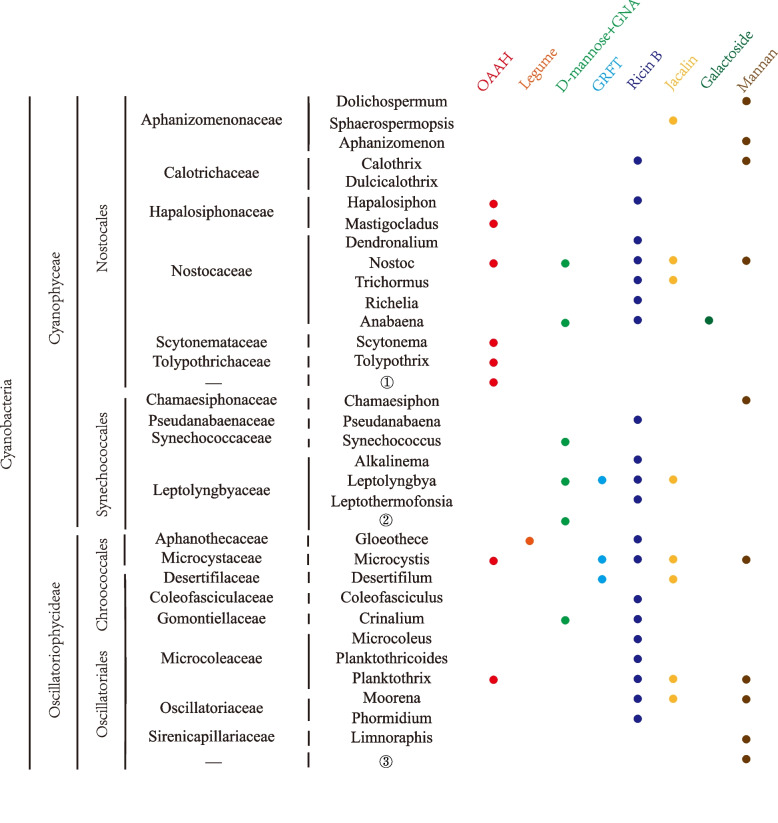
Coulson plot of lectin domains across a range of cyanobacteria. The left panel shows the classification of cyanobacteria. ①②③ represent three unclassified species (GenBank accesssions ARV63087.1, EKQ69920.1, TAE43472.1) in their respective taxonomical groups and the “-” symbols indicates their unclassified taxonomical levels. The different colored dots on the right panel indicate presence or absence of genes from corresponding lectin families

Multiple lectin families have been reported in plant according to different taxonomic approaches, each with a specific carbohydrate recognition domain. The results of the HMMER similarity search showed that 105 lectins were classified out of 196 lectins, and seven of them belonged to two lectin family (one each from *Crinalium*, *Desertifilum* and *Leptolyngbya*, and four from *Microcystis*), as shown in Fig. 1. They may take into account the properties of both families. This phenomenon may provide a new direction for the development of lectins. As shown in Fig. 2, the existing lectins are primarily distributed in the orders Nostocales, Oscillatoriales, Chroococcales and Synechococcales, involving both unicellular and multicellular filamentous cyanobacteria. In Nostoc of the Nostocales and Microcystis of the Chroococcales, five lectin families are involved, which may be strongly related to the size of their genomes. The lectin families in these genomes are mainly dominated by the Ricin B and Mannan-binding lectin families and are distributed in all four cyanobacterial orders. And no MFP-2, Chitin, and Nictaba family lectins were found in the sequenced lectins of cyanobacteria This suggests that the origin and evolution of these three lectin families may not be related to cyanobacteria.

Using the classification results of lectin families obtained above, a local blast database was established. The CDS sequences of cyanobacterial genes have been searched for possible lectin sequences in cyanobacteria using TBLASTN 2.9.0 + . This will aid in the development of lectins and provide guidance for the discovery of new lectins. As shown in Table 1, the types of lectins mined were mainly Ricin B and Jacalin lectin families, and the same as above, MFP-2, Chitin and Nictaba family lectins were not found, and the lectins were mostly concentrated in Nostocales, Oscillatoriales and Synechococcales. In addition, the results reveal the possibility of the presence of lectins in five other cyanobacterial orders, and it is also evident that none of the examined lectin families are present in the cyanobacterial orders Gloeomargartales and Thermosticales (verified by online BLAST).

**Table 1:** Taxonomic distribution and quantity of lectin domains among cyanobacterial orders

	**OAAH**	**Legume**	**D-mannose+GNA**	**MFP-2**	**GRFT**	**Ricin B**	**Jacalin**	**Galactoside**	**Chitin**	**Nictaba**	**Mannan-binding**
Chroococcales	—	2	4	—	6	12	8	4	—	—	—
Chroococcidiopsidales	—	—	—	—	—	1	—	—	—	—	—
Desulfuromonadales	—	—	1	—	—	1	1	—	—	—	—
Gloeobacterales	—	2	—	—	—	—	—	1	—	—	—
Gloeomargaritales	—	—	—	—	—	—	—	—	—	—	—
Nostocales	5	8	19	—	—	59	45	18	—	—	2
Oscillatoriales	1	5	18	—	—	48	38	8	—	—	3
Pleurocapsales	—	1	2	—	—	3	2	—	—	—	—
Spirulinales	—	1	5	—	—	8	1	2	—	—	—
Synechococcales	—	7	22	—	2	60	24	6	—	—	1
Thermostichales	—	—	—	—	—	—	—	—	—	—	—

The “-” symbols indicates "not found". Source of the genome sequence data is provided as supplementary material in Table S1

### Evolution and motif patterns of lectins in cyanobacteria

The evolution of the Legume lectin-like lectin family, the Griffithsin lectin family, the OAAH lectin family and the Mannan-binding lectin family were analyzed to understand the evolution of the cyanobacterial lectins and to provide information for discovery of new lectin.

**Fig. 3:**
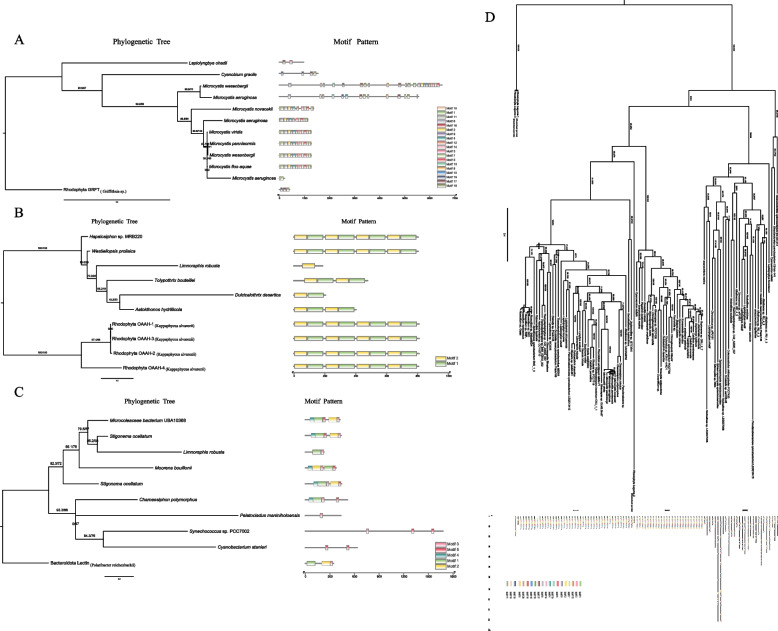
Maximum likelihood phylogenies and motif patterns of four lectin families in cyanobacteria. **A** GRFT lectin family, with Rhodophyta GRFT from *Griffithsia* sp. (GenBank accession AY744144.1) used as the out-group. **B** OAAH lectin family, with four homologous sequences from *Kappaphycus alvarezii* (numbered as Rhodophyta OAAH-1–4) used as the out-group; **C** Mannan-binding lectin family, with the Bacteroidota lectin sequence from *Polaribacter reichenbachii* used as the out-group; **D** Legume lectin-like lectin family, with homologous sequences from *Rhodochaete parvula* (numbered as Rhodophyta legume 1–3) used as the outgroup.. The number pairs on the branches represent both SH-aLRT test and bootstrap values with 1000 replicates. Accession numbers for the sequences data are provided as supplementary Table S1

Griffithsin homologs in cyanobacteria are rare and exhibit distinct motif patterns (Fig. 3A). And notably, there are both scattered and compact motif patterns in *Microcystis wesenbergii* and *Microcystis aeruginosa*. Take into consideration that red algal GRFT shows excellent anti-viral and anti-tumor activities, it worth an attempt to check if some cyanobacterial lectins with similar motifs, such as those from *Cyanobium gracile*, *Microcystis wesenbergii* and *Microcystis aeruginosa*, have similar activities.

The sequence length of OAAH family lectins is generally short. And not only that, as one can see from the Fig. 3B, their sequences are repetitive, which also confirms the previous report that their sequences have four or two repeats [19]. The first OAAH family lectin has been reported as a two repeat type from the caynobacterium *Planktothrix* (the former name, *Oscillatoria*) *agardhii*, and later Rhodophyta lectins with four repeats were found out [30]. This special structure is closely related to their functions, as shown in Fig. 3B, *Limnoraphis robusta* has no complete structure may not contain OAAH family lectins, and *Dulcicalothrix desertica,* contains a functional domain, which may be a novel type of OAAH family. The most representative lectin of the Mannan-binding family is MVL, which is only found in bacteria, reflecting the bacterial properties of cyanobacteria. The motif pattern of lectins in *Synechococcus* sp. PCC7002, *Pelatocladus maniniholoensis* and *Cyanobacterium stanieri* is more concise (Fig. 3C). This will be more favorable for the synthesis and expression of the lectin as well as the mechanism study. The Legume lectin-like lectin family is widely distributed in cyanobacteria, with the most results founded. This lectin family can be roughly classified into three subgroups in cyanobacteria, Legume-I, Legume-II and Legume-III (Fig. 3D). The main motifs of Legume-I are 1, 3, 12, 7, 2, 9, 5, 13, 4, 14, and 6, arranged in this order. The main motifs of Legume- II are motifs 10, 1, 3, 18, 6, 2, 9, 5, 4, 14, and 8. Legume III is mostly 17, 18, and 15, 16. And there are also replicates of motifs 17 and 18. The motif arrangements of the subgroups Legume-I and Legume-II are compact, and the patterns between the two subgroups are different. Whereas the motif pattern in the subgroup Legume I shows very scattered in arrangement and distinct in composition. Legume I has a unique motif ranking of 12, 7. Legume II has two unique motifs, 18 and 8. Legume III has a unique repetition phenomenon. Subgroups Legume I and II form a clade which is relatively distant from the subgroup Legume III. Legume-Ι is founded in two orders more than Legume-II, Spirulinales and Pleurocapsales, and Legume-III is founded in one more order of Gloeobacterales than Legume-II. In addition, with three homologous Legume lectin-like lectin sequences from red algae *Rhodochaete parvula* (numbered as Rhodophyta Legume-1 to 3) as the outgroup (Fig. 3D), Rhodophyta Legume-2 is encompassed by cyanobacterial lectins and is distant from the other two homologs. And the reason for this phenomenon may be due to horizontal transfer of genes. Phylogeny of the three homologs implies the origins of plant lectins both from the ancient host and cyanobacteria by endosymbiotic gene transfer during the early evolution of Archaeplastida.

## Conclusion

At present, most of the research on cyanobacterial lectins is focused on the in vitro expression and synthesis, and the development of new cyanobacterial lectins is insufficient, and there is blindness to the activity and function of the obtained cyanobacterial lectins. The research helps provide the basis for the discovery and activity study of cyanobacterial lectins.

Seven lectins have been compared and found to combine the structural domains of two different lectin families, and they may also combine the properties of both families in terms of functional activity. MFP-2, Chitin and Nictaba lectin families were not found in cyanobacteria. Eight lectin families showed some deficiencies in the distribution of cyanobacteria, which could be due to the transfer of genes in the evolutionary process caused by the genes, or possibly due to the transfer of horizontal genes. In addition, the evolutionary tree and motif analysis showed that the Griffithsin lectin family may have evolved from *Microcystis* in cyanobacteria, and the lectins contained in the Griffithsin family are currently found only in red algae, but have good medicinal value, so the discovery of the Griffithsin lectin family in cyanobacteria has great significance for the study of this family. In cyanobacteria, the OAAH family was also found, with two or four repetitive sequences like the previously reported, but also a single sequence without duplication, which may provide a new direction for the study of this family. The Legume lectin-like lectin family is the most widely distributed and numerous in cyanobacteria, and gene horizontal transfer may happened in this family.

## Methods

### Compilation of lectin homolog datasets

In this study, all 12 known lectin families found so far in plant, algae and cyanobacteria were examined, and they were grouped according to different classifications such as type of sugar binding, structure, and molecular backbone, and each lectin family has different properties. For example, in the Griffithsin lectin family, there is currently only one GRFT lectin obtained from the red alga *Griffithsia* sp. which has excellent antiviral and antitumour effects. Or the OAAH family, Oscillatoria agardhii lectin homolog (OAAH) lectin family, which has a beta-barrel scaffold [18], generally divided into two types of four repeats and two repeats [19].

The summarized lectin family includes: Chitin lectin family, D-mannose lectin family, GNA-like lectin family, Galactoside lectin family, Griffithsin lectin family, Jacalin lectin family, Legume lectin-like lectin family, MFP-2 lectin family, Nictaba lectin family, OAAH lectin family, Ricin B lectin family, Mannan-binding lectin, among which D-mannose lectin family and GNA-like lectin family have the same structural domain, so it can be concluded that they may be the same class of lectins. Except for the Griffithsin and MFP-2 lectin families, all other lectin families have Pfam identifiers, PF00187 (Chitin lectin family), PF01453 (D-mannose and GNA-like lectin family), PF00337 (Galactoside lectin family), PF01419 (Jacalin lectin family), PF03388 (Legume lectin-like lectin family), PF14299 (Nictaba lectin family), PF17882 (OAAH lectin family), PF14200 (Ricin B lectin family), PF12151 (Mannan-binding lectin).

A total of 196 cyanobacterial lectin sequences were collected from 25 families and 51 genera. And 319 cyanobacterial CDS sequences and protein sequences were selected from 51 families and 146 genera.

The cyanobacterial lectin sequences were compared using HMMER 3.3.2. For the Griffithsin and MFP-2 lectin families with no reported recognition domains, BLAST 2.9.0 + was used to mine the distribution of each family among the existing cyanobacterial lectins. A tblastn local search comparing cyanobacterial CDS sequences was used to explore the distribution of each cyanobacterial lectin family in cyanobacteria.

### Phylogenetic analysis of lectins

To understand the evolution of the Legume lectin-like lectin family, the Griffithin lectin family, the OAAH lectin family and the Mannan-binding lectin family, an evolutionary tree was constructed using IQ-TREE [31]. Homologous lectin sequences from red algae were used as outgroups (Table S3). The Mannan-binding family was introduced as an outgroup to the same family of bacterial lectins, as they only exist in bacteria.

### Motif pattern analysis

MEME 5.5.1 was used to perform the analysis online [32]. Among the above identified sequences of the structural domains of the lectin families, the conserved motifs were excavated. The parameters were set as follows: classical model, size of the motifs 6–100/6–50. The distribution of the selected significant motifs in the sequences and between species was analyzed.

### Supplementary Information

**Additional file 1:** **Table S1.** Selected cyanobacterial genome assemblies from GenBank and the taxonomy of the source organisms.

**Additional file 2:** **Table S2.** Available cyanobacterial lectin sequences and the taxonomy of the source organisms in Genbank. Experimentally identified sequences are shown with name in the Name column.

**Additional file 3:** **Table S3.** Rhodophyta lectin sequences used as outgroups in Fig. 3. Note that the OAAH and Legume family sequences were orignally from transciptome data of NCBI SRA database which were assembled in this study.

## Data Availability

The datasets analysed during the current study are available in the Figshare repository, https://figshare.com/s/89657efa2a9c65c2475c and all the accession numbers of the sequences are provided in supplementary Tables S1 and S2 which can also be downloaded from NCBI GenBank https://www.ncbi.nlm.nih.gov/.
